# The Lactate Minimum Test: Concept, Methodological Aspects and Insights for Future Investigations in Human and Animal Models

**DOI:** 10.3389/fphys.2017.00389

**Published:** 2017-06-08

**Authors:** Leonardo H. D. Messias, Claudio A. Gobatto, Wladimir R. Beck, Fúlvia B. Manchado-Gobatto

**Affiliations:** ^1^School of Applied Sciences, University of CampinasSao Paulo, Brazil; ^2^Department of Physiological Sciences, Biological and Health Sciences Center, Federal University of São CarlosSão Paulo, Brazil

**Keywords:** lactate minimum intensity, maximal lactate steady state intensity, anaerobic threshold, individualized exercise prescription, protocol validity

## Abstract

In 1993, Uwe Tegtbur proposed a useful physiological protocol named the lactate minimum test (LMT). This test consists of three distinct phases. Firstly, subjects must perform high intensity efforts to induce hyperlactatemia (phase 1). Subsequently, 8 min of recovery are allowed for transposition of lactate from myocytes (for instance) to the bloodstream (phase 2). Right after the recovery, subjects are submitted to an incremental test until exhaustion (phase 3). The blood lactate concentration is expected to fall during the first stages of the incremental test and as the intensity increases in subsequent stages, to rise again forming a “U” shaped blood lactate kinetic. The minimum point of this curve, named the lactate minimum intensity (LMI), provides an estimation of the intensity that represents the balance between the appearance and clearance of arterial blood lactate, known as the maximal lactate steady state intensity (iMLSS). Furthermore, in addition to the iMLSS estimation, studies have also determined anaerobic parameters (e.g., peak, mean, and minimum force/power) during phase 1 and also the maximum oxygen consumption in phase 3; therefore, the LMT is considered a robust physiological protocol. Although, encouraging reports have been published in both human and animal models, there are still some controversies regarding three main factors: (1) the influence of methodological aspects on the LMT parameters; (2) LMT effectiveness for monitoring training effects; and (3) the LMI as a valid iMLSS estimator. Therefore, the aim of this review is to provide a balanced discussion between scientific evidence of the aforementioned issues, and insights for future investigations are suggested. In summary, further analyses is necessary to determine whether these factors are worthy, since the LMT is relevant in several contexts of health sciences.

## Introduction

The important role of individualized exercise intensity has been accepted worldwide in several areas of the health sciences. These include athlete's performance improvements (Jones and Carter, 2000; Bosquet et al., 2002; Bentley et al., 2007), non-athletes' health (Mendes et al., 2015; Parmenter et al., 2015) and also patients with cancer, cardiovascular disease, diabetes and other diseases, in order to improve their clinical status and quality of life (Wasserman and McIlroy, 1964; Paffenbarger and Hyde, 1984; Klika et al., 2009; Kirkham et al., 2013; O'Hagan et al., 2013; Scharhag-Rosenberger et al., 2015). Irrespective of the context in which exercise and its individualized intensity prescription are used, researchers constantly focus on the accurate determination of variables used to prescribe the exercise intensity. In this sense, the search for tests or protocols that provide reliable and reproducible results is a key factor for training effectiveness, including its prescription, and exercise control.

Physiological parameters such as the percentage of maximal heart rate (HR_max_) (Karvonen and Vuorimaa, 1988), maximal oxygen consumption (VO_2max_) (Poole and Richardson, 1997) and metabolic markers like the blood lactate concentration ([Lac]) (Billat, 1996) have been extensively used for individualized exercise prescription. Although, the prescription of relative or absolute intensities has demonstrated high applicability for many populations, criticism exists around the physiological tests or protocols used to assess cardiovascular, respiratory, and metabolic variables. Due to these criticisms, exercise physiologists requested new tests or studies and improvements to tests that have been already proposed.

In this way Uwe Tegtbur, professor and researcher at Hannover Medical School, proposed the named lactate minimum test (LMT) in the early 90s (Tegtbur et al., 1993). This test begins with high intensity efforts to induce a hyperlactatemia state (phase 1), followed by 8 min of passive rest (phase 2) and finally, an incremental test initialized with low exercise intensity but high blood lactate concentration (phase 3). During the first incremental stages, the blood lactate concentration is expected to fall into a minimum point and rise again over the following stages. According to Tegtbur et al. (1993) this minimum point named the lactate minimum intensity (LMI), provides an estimation of the intensity that represents the balance between the appearance and clearance of arterial blood lactate, known as the maximal lactate steady state intensity (iMLSS). Apart from the aerobic capacity estimation, studies have also applied anaerobic protocols during the hyperlactatemia phase (Smith et al., 2002; Araujo et al., 2013; Camargo et al., 2013; Messias et al., 2015), allowing the determination of anaerobic indexes, such as peak, mean and minimum force/power. Therefore, in addition to its easy application and objectivity, the LMT is also considered a valid physiological protocol for the assessment of aerobic and anaerobic parameters in just one evaluation session (Messias et al., 2015).

Over the years, the LMT has gained popularity around the world, and its application has been seen in several contexts including studies with diabetes (de Oliveira et al., 2007; Oliveira et al., 2010), thrombocytosis (Beck et al., 2014b) and wheelchair-racing athletes (Perret et al., 2012). Despite the fact that important concerns were arisen so far, some questions remain requiring discussion. Can this test be influenced by methodological aspects related to the hyperlactatemia induction, recovery and incremental exercise? Evidence has drawn opposite conclusions regarding the influence of the hyperlactatemia induction modes on determination of the LMT parameters (Smith et al., 2002; Zagatto et al., 2014). These controversies require a balanced discussion of the topic, since its necessity during the LMT is indispensable (see explanation on the next section). Along with the hyperlactatemia modes, some attention has been given to the recovery type and length during phase 2 (Ribeiro et al., 2009). The mitigating factor around this issue is related to a myriad of recovery manipulation used by studies involving this test. The discussion in this topic is required and overdue since its influence on LMT parameters was demonstrated (Ribeiro et al., 2009). Opposite conclusions are also shown regarding the incremental test initiated with high blood lactate concentration (Carter et al., 1999b; Ribeiro et al., 2003). This topic is of utmost relevance, since the determination of LMT parameters is guided by the blood lactate concentration, which can be affected by the stage's intensity (Bentley et al., 2007) and length (Yoshida, 1984).

Along with the methodological controversies, two other points must be better explored. Is the LMI a valid parameter for monitoring longitudinal training effects? Here, we have a favorable discrepancy regarding the LMI effectiveness (Silva et al., 2007; Miranda et al., 2013; Campos et al., 2014); however, this perspective has hardly been criticized (Carter et al., 1999a) and a balanced discussion around both sides is required. The last and debatable point is related to the relationship between the iMLSS and LMI. Does the LMI indeed estimate the iMLSS? This issue can be considered as one of most studied (Jones and Doust, 1998; MacIntosh et al., 2002; Johnson et al., 2009; Dotan et al., 2011; Knoepfli-Lenzin and Boutellier, 2011), since the MLSS protocol is considered the gold standard protocol for the anaerobic threshold estimation (Heck et al., 1985; Beneke, 1995, 2003; Beneke and von Duvillard, 1996; Beneke et al., 1996, 2000). Additionally, despite the relationship between the iMLSS and LMI is the key aim of the LMT proponents (see next section), there are controversies regarding this issue. Moreover, important methodological concerns were not considered when the comparison between these two intensities was made. These subjects will be discussed in this review.

We have listed questions that still require deep discussion. These points are important for both science and practice and a balanced discussion can help further studies to improve the knowledge around the LMT. Therefore, the main aim of this review is to provide a balanced discussion between studies that are favorable to the LMT with those that criticized this test, especially regarding the listed points that still require investigation. There are four specific objectives of this review: (a) to discuss the possible influence of the hyperlactatemia induction, recovery and incremental test on determination of the LMT parameters; (b) to debate the factors that are impairing the LMI effectiveness for monitoring training effects, especially those applied to improve aerobic parameters (i.e., aerobic power and capacity); (c) to examine studies that compared the LMI with iMLSS and discuss possible factors that still hamper the relationship between these two intensities; and (d) transpose all the discussed perspectives to animal model.

Firstly, a historical perspective about the LMT will be provided. This is necessary to understand how this test was proposed and why its parameters have physiological significance. Then, in the section “Can the LMT be influenced by methodological aspects?” the controversies related to the LMT phases will be discussed and insights about future investigations will be provided. The section “Is the LMI modified after training?” refers to controversies regarding the LMI effectiveness for monitoring training effects. We propose an untested hypothesis that relates this issue with a possible mathematical modeling dependency. Subsequently, studies that compared the LMI with iMLSS will be discussed in the section “Does the LMI indeed estimate the iMLSS?.” As in the previous section, we propose another untested hypothesis to improve the knowledge around the relationship between these two intensities; this perspective is related to methodological concerns of both protocols, especially in the determination of iMLSS. Lastly, considering this test is not limited to the human model, we included a section that discusses every controversial point highlighted so far in animal models.

Relevant literature was considered by searching PubMed, SPORTDiscuss, Scopus, SCIELO, and Google Scholar databases. Studies were included based on the aims of this review. Therefore, queries comprising combinations of terms such as “lactate minimum test,” “hyperlactatemia,” “recovery,” “incremental test,” “methodological aspects,” “training,” “lactate minimum intensity,” “anaerobic threshold,” and “maximal lactate steady state intensity” were considered. We also accessed studies referenced by any of the already published papers. English language studies were included in this review without any restriction on publication date. Sample characteristics, reproducibility of the results showed in the cited studies and the numbers of participants evaluated were not limiting factors and did not determine the study inclusion. The standardized mean difference (SMD) between the results of the studies was calculated to provide practical inferences and support the study conclusions. The comparison of data from two groups was performed using SMD = (first group mean data—second group mean data) / standard deviation average from both groups. When studies compared more than two groups, the SMD was calculated as SMD = (highest mean data—lowest mean data) / square root of the mean square error.

## Theoretical concept of the LMT

In the field of exercise physiology, it is necessary to understand which factors allow the body to continue exercising. In the first chapter of his book, Mosso (1904) provides an interesting discussion about the migration of birds and how the “fatigue signals” are evident over their journey, mainly when they arrive to the desired location. In isolated muscles, research published at the beginning of the twentieth century showed that the lactic acid, previously discovered in 1780 in sour milk by Carl Wilhelm Scheele, is also produced during muscle metabolic processes (for complete historical background see Gladden, 2008). In 1923, an article by Hill and Lupton entitled “*Muscular exercise, lactic acid, and the supply and utilization of oxygen*” was the first published work to suggest that lactic acid reflects the exercise intensity, and its production is dependent upon the muscle oxygen supply (Hill and Lupton, 1923).

Even with fewer technological possibilities, Hill and Lupton importantly stated that lactic acid production does not solely occur in the oxygen absence, but there is also a balance between its production and removal at rest (Hill and Lupton, 1923). The possibility that a “physiological steady state” exists in some exercise intensities and that this steady state may be estimated by the lactate concentration in the blood stream were novel insights. Ten years later, Margaria et al. (1933) suggested, based on several experiments of high intensity exercises (i.e., 4–10 min), that blood lactate accumulation was not temporally associated with the post-exercise oxygen consumption, proposing the term “alactacid.” Despite this novel finding, it is clear that in Margaria's article the steady state was already considered an important phenomenon. These authors precisely stated that in exercise (~10 min) not involving the maximum metabolic rate, there is a balance between the lactate production and diffusion from muscles into the blood stream, and the diffusion from the blood stream to tissues that are not producing lactate.

It is valid to state that authors have challenged the post-exercise oxygen debt theory (Gaesser and Brooks, 1984; Brooks, 1985). Apart from the controversies, the fact is that several studies contributed significantly to the understanding of possible mechanisms of the steady state phenomenon in exercise (Bang, 1936; Wasserman and McIlroy, 1964; Kindermann et al., 1979; Sjodin and Jacobs, 1981; Stegmann et al., 1981; Heck et al., 1985; Mader and Heck, 1986; Gobatto et al., 2001). For instance, the hyperbolic power-time relationship from the critical power concept (Monod and Scherrer, 1965) aims to determine the highest intensity that may be sustained for a long time without fatigue (for revision see Jones et al., 2010). The point of optimal respiratory efficiency (Hollmann, 1959), onset of blood lactate accumulation (Sjodin and Jacobs, 1981) and the so called thresholds concepts (Wasserman and McIlroy, 1964; Kindermann et al., 1979) were also proposed with basis in the steady state phenomenon. Regarding the thresholds concepts, Wasserman and McIlroy (1964) proposed that during an incremental cycle ergometer exercise an “anaerobic threshold” may be detected based on the respiratory gas exchange ratio (i.e., the ratio between CO_2_ production to O_2_ consumption). The anaerobic threshold detection was originally proposed to avoid exhaustive and potentially dangerous exercise for cardiac patients. According to these authors, this phenomenon is defined as the onset of significant anaerobic metabolism during exercise.

Conversely, Kindermann et al. (1979) challenged the concept based on a second exponential rise of the blood lactate concentration during the latter stages of an incremental treadmill exercise. Then, the former concept stated by Wasserman and McIlroy (1964) proposed the first elevation of blood lactate (~2 mmol.l^−1^) concomitantly with a non-linear increase of the ventilation and respiratory quotient, named the “aerobic threshold” (AT). On the other hand, the anaerobic threshold (AT_2_) was considered the steep part of the exponential rise in blood lactate. The anaerobic threshold concept (i.e., AT_2_) will be used throughout this review.

Considering the great utilization of incremental tests for AT determination, Davis and Gass (1979) importantly showed whether the blood lactate is elevated prior to the incremental exercise the lactate kinetic is affected. It is clear from Davis and Gass (1979) study that there is a difference in the blood lactate curve during the incremental test of Test 3. Since the lactate concentration reduction in Test 3 occurred at levels far above the AT determined in Tests 1 and 2, Davis and Gass (1979) confirmed the AT_2_ concept at the point in which the blood lactate concentration increased significantly in the latter test. According to these authors, the range between AT and AT_2_ predicts a steady state work intensity. Another interesting fact was that in every test the blood lactate concentration increased at ~260 W. In fact, this increase was expected, since its production and clearance are unbalanced at intensities above the AT_2_. However, considering that in Test 3 the subjects began the incremental exercise with a high blood lactate concentration, the blood lactate firstly decreased at the 6th min into a minimum point, and then increased again until the test ended.

Fourteen years after the Davis and Gass article, Tegtbur et al. (1993) turned their attention to the “minimum point.” The first working hypothesis of these authors was that the minimum point would represent a maximal intensity where the blood lactate production and catabolism are in balance, which is the iMLSS. Additionally, by relying on Poole's study (Poole et al., 1988), these authors proposed the second working hypothesis, which was that the minimum point would correspond to the power asymptote (Moritani et al., 1981) from the power-time relationship (Monod and Scherrer, 1965).

To test these hypotheses, endurance-trained runners (ER) and less endurance trained basketball players (BB) were evaluated on a 200 m indoor track under the same method. The LMT was then proposed using three phases: (1) an initial lactic acidosis induction phase (i.e., hyperlactatemia phase) based on two exhaustive efforts of 300 and 200 m for ER, or 2 × 200 m for BB with 1 min recovery; (2) an 8 min recovery allowing the lactate to diffuse from the muscles to the blood stream; and (3) an incremental test with an initial speed ranging from 3.00 to 4.33 m.s^−1^, with increases in the running speed by 0.33 m.s^−1^ every 800 m until exhaustion.

It is possible to notice from the study of Tegtbur et al. (1993) the “U” shaped form of the blood lactate concentration during the incremental test. As previously noted the blood lactate concentration is expected to fall into a minimum point and then rise again until exhaustion due to the low intensity of the first incremental stages. The lactate minimum speed (LMS; i.e., LMI in terms of running exercise) was considered to be the intensity (x axis—vertical line) related to the minimum point in the “U” shaped curve and, according to the authors, identified by a spline function. Considering the first hypothesis, the LMS should represent an intensity in which the blood lactate production and catabolism are in balance.

Aiming to test this hypothesis, the subjects completed two 8 km constant load runs at the LMS and at 0.2 m.s^−1^ above the LMS. The authors showed that only a 0.2 m.s^−1^ increase in the LMS also resulted in a significant increase of 1.9 ± 1.1 mmol.l^−1^ in blood lactate concentration. However, no significant increase was found during the 8 km run at LMS (i.e., 0.4 ± 0.4 mmol.l^−1^). Despite the fact that the MLSS protocol was not properly applied (i.e., the subjects only ran in two different intensities), the authors accepted the first hypothesis by concluding that the LMS and iMLSS were equal. Moreover, considering that Poole et al. (1988) successfully estimated the iMLSS by using the hyperbolic power-time relationship, Tegtbur and colleagues also accepted the second working hypothesis, even without comparing the asymptote from the power-time relationship and the LMS. Considering these results, the authors proposed the LMT as an easy alternative protocol for estimating the steady state phenomenon during exercise, since the MLSS and the critical power test may take at least 4–5 days and are, therefore, inappropriate for routine use.

Despite this important result, the authors also investigated whether the LMS is a function of the incremental test duration and is also dependent on the muscle glycogen content. Regarding the incremental test duration, it was demonstrated that the LMS would not be changed whether the 800 m or 1200 m tests were applied (LMS = 4.49 ± 0.17 m.s^−1^ and 4.44 ± 0.18 m.s^−1^, respectively). However, high LMS (4.96 ± 0.35 m.s^−1^) was obtained when 400 m were used (400 vs. 800 m *P* ≤ 0.001; 400 vs. 1,200 *P* ≤ 0.001). Thus, the authors suggested that both 800 m and 1200 m could be used in the incremental test, but considering practical reasons the 800 m should be chosen. It was also shown that the muscle glycogen storage did not influence the LMS determination, since a mean difference of 0.00 ± 0.09 m.s^−1^ was obtained between a group that had 1 day rest plus a carbohydrate-rich diet before the LMT and a group that performed an exhaustive high-intensity run of 80 min followed by a 24 h low carbohydrate diet.

After its proposal, the LMT was further recognized as a valid physiological protocol, especially in terms of anaerobic threshold estimation (MacIntosh et al., 2002; Johnson et al., 2009). However, the comparison with other graded exercise tests (GXT; e.g., Balk or Bruce) is unavoidable. The LMT is actually an alternative to estimate the anaerobic threshold and it is difficult to impose huge differences between its application and GXT, since in both cases an incremental test is applied. On the other hand, some LMT advantages can be suggested: (a) the necessity for hyperlactatemia induction permits that other tests such as the Wingate anaerobic test (Bar-Or et al., 1977) or running anaerobic sprint test (Zacharogiannis et al., 2004) might be applied in this phase, allowing the determination of anaerobic indexes (e.g., peak, mean, and minimum power/force). This possibility is not present in applications of GXT; (b) the LMI (e.g., LMS) is individual and it is not associated with fixed blood lactate concentration (Heck et al., 1985); and (c) although mathematical functions (e.g., segmentation of lines along with intersection determination; Hinkley, 1969) were proposed to identify the individual anaerobic threshold during GXT (Stegmann et al., 1981), the LMI identification is easier since the simple visual inspection seems consistent with mathematical functions (e.g., cubic spline of polynomial functions; Ribeiro et al., 2003; Dotan et al., 2011).

Nowadays, the LMT is used in several contexts in which exercise or physiological evaluations are required. For instance, its application was already conducted in a specific manner for taekwondo (Mota et al., 2011), basketball (Araujo et al., 2013; Camargo et al., 2013), table tennis (Barbieri and Gobatto, 2013), triathlon (Vicente-Campous et al., 2014), and futsal (Messias et al., 2015; Barbieri et al., 2017) besides several other important issues (Carter et al., 2000; Davison et al., 2000; Dantas De Luca et al., 2003; Zagatto et al., 2004, 2013; Simões et al., 2005; Rotstein et al., 2007; Mezzaroba and Machado, 2013; Miyagi et al., 2015; Sousa et al., 2017). Along with its popularity, concerns have arisen involving the influence of possible methodological aspects on LMT parameters, and effectiveness of LMI for identifying training effects or even iMLSS estimation validity. In the next sections a balanced discussion of these topics will be provided. Additionally, future insights will be suggested in order to improve the scientific knowledge of this test.

## Can the LMT be influenced by methodological aspects?

One of the greatest LMT concerns is the potential influence that the methodological aspects may have on the variables obtained by this test, especially the LMI. As previously noted, the LMT is composed of three phases, making this test potentially dependent on protocol manipulations (Ribeiro et al., 2009). Thus, studies have aimed to investigate whether the hyperlactatemia induction (Smith et al., 2002; Johnson et al., 2009; Zagatto et al., 2014), recovery (Denadai and Higino, 2004; Ribeiro et al., 2009) and the incremental exercise (Carter et al., 1999b; Ribeiro et al., 2003; Sotero et al., 2007; Pardono et al., 2008; Johnson and Sharpe, 2011; Miyagi et al., 2013) may affect the LMI determination (Table 1).

**Table 1:** Characterization of studies with humans that investigated methodological aspects related to the LMI determination in terms of hyperlactatemia induction (phase 1), recovery (phase 2), and incremental exercise (phase 3).

**Study authors/country**	**Study population (n°/training level/gender)**	**LMT phase/study aim^§^**	**Test protocols**	**Study results (LMI)**	**Study conclusion^§§^**
Smith et al., 2002/United Kingdom–Australia.	8/athletes-cyclists/non-specified.	1/to investigate the effects of four hyperlactatemia induction protocols on LMI determination.	Hyperlactatemia protocols: (i) ramp test; (ii) 30-s maximal sprint; (iii) 40-s maximal sprint; (iv) double 20-s maximal sprint with 60-s active recovery between sprints.	(i) 298.0 ± 15.0; (ii) 293.0 ± 15.0; (iii) 293.0 ± 16.0; (iv) 296.0 ± 18.0 W. *P* = 0.275; SMD-0.120.	The LMI determination was not dependent upon the hyperlactatemia induction protocols.
Johnson et al., 2009/United Kingdom.	10/active/male.	1/to investigate the effects of muscle groups used during two hyperlactatemia induction protocols on LMI determination.	Hyperlactatemia protocols: (i) GXT performed in cycle ergometer; (ii) GXT performed in arm-cranking ergometer.	(i) 168.0 ± 21.0; ii) 157.0 ± 29.0 W. *P* < 0.05^#^; SMD-0.440.	The LMI determination is influenced by the muscle groups used during the hyperlactatemia induction.
Zagatto et al., 2014/Brazil–Italy.	20/recreationally trained (*n* = 15) and triathletes (*n* = 5)/male.	1/to investigate the effects of three hyperlactatemia induction protocols on LMI determination.	Hyperlactatemia protocols: (i) GXT; (ii) 30-s maximal sprint; (iii) double 30-s maximal sprint with 30-s passive recovery between sprints.	(i) 187.3 ± 31.9; (ii) 189.8 ± 26.0; (iii) 200.3 ± 25.8 W. *P* = 0.000 (i and ii ≠ iii); SMD-0.448.	The LMI determination is affected by the hyperlactatemia induction protocols.
Denadai and Higino, 2004/Brazil.	25/sprinters (*n* = 13) and endurance runners (*n* = 12) athletes/non-specified.	2/(a) to investigate the effects of individualized or non-individualized recovery on phase 2; (b) to determine possible differences between endurance runners and sprinters under the above mentioned conditions.	Recovery protocols: (i) non-individualized recovery (8-min) for endurance runners; (ii) individualized recovery for endurance runners; (iii) non-individualized recovery (8-min) for sprinters; iv) individualized recovery for sprinters.	(i) 285.7 ± 19.9; (ii) 283.9 ± 17.8; (iii) 238.0 ± 14.1; (iv) 239.4 ± 13.9 m min^−1^. *P* < 0.05^#^ (i and ii ≠ iii and iv); SMD-2.700.	(a) The LMI determination seems not to be influenced by passive recovery applied under individualized or non-individualized condition. (b) This condition did not influenced on LMI of endurance and sprinter runners (i = ii; iii = iv).
Ribeiro et al., 2009/Brazil.	12/trained runners/male.	2/to investigate the influence of using different recovery intensities during phase 2.	Recovery protocols: (i) 8-min passive recovery; (ii) 8-min active recovery at 30% of the VO2_max_ velocity; (iii) 8-min active recovery at 50% of the VO2_max_ velocity.	(i) 13.8 ± 1.6; (ii) 13.3 ± 1.6; (iii) 12.8 ± 1.5 km.h^−1^. *P* < 0.05^#^(i ≠ ii and iii; ii ≠ iii); SMD-0.644.	The LMI determination seems to be dependent on the recovery intensity applied in phase 2.
Carter et al., 1999b/United Kingdom.	8/trained runners/male.	3/to investigate the effects of manipulating the initial intensity in the phase 3.	Initial intensity protocols: (i) 3.0 km.h^−1^below AT; (ii) 2.5 km.h^−1^ below AT; (iii) 2.0 km.h^−1^ below AT; (iv) 1.5 km.h^−1^ below AT; (v) 1.0 km.h^−1^ below AT; (vi) 0.5 km.h^−1^ below AT; (vii) at AT; (viii) 1.0 km.h^−1^ above AT.	(i) 13.8 ± 0.7; (ii) 14.5 ± 0.6; (iii) 14.5 ± 0.7; (iv) 15.0 ± 0.8; (v) 15.1 ± 0.6; (vi) 15.8 ± 0.8 km.h^−1^; (vii) non-determined; (viii) non-determined. *P* < 0.05^#^(i ≠ all; vi ≠ all; ii ≠ all but iii; iii ≠ all but ii; iv ≠ all but v; v ≠ but iv); SMD-2.660.	The LMI determination is affected whether different initial intensities are employed in phase 3.
Ribeiro et al., 2003/Brazil.	12/trained swimmers/male.	3/(a) to investigate the effects of using different stage lengths during phase 3; (b) to investigate the LMI determination using mathematical function or visual inspection.	Stage lengths within mathematical analysis: (i) stages of 200 m using visual inspection; (ii) stages of 200 m using spline function; (iii) stages of 300 m using visual inspection; (iv) stages of 300 m using spline inspection.	(i) 1.31 ± 0.12; (ii) 1.32 ± 0.10; (iii) 1.28 ± 0.11; (iv) 1.28 ± 0.10 m.s^−1^. *P* > 0.05^#^; SMD-0.400.	(a) The LMI determination is not affected by the used stage lengths; (b) LMI determination is also not affected whether mathematical function or visual inspection are used.
Sotero et al., 2007/Brazil.	17/active/male.	3/(a) to investigate the effects using all or only three incremental stages for LMI determination; (b) to investigate the effects of using mathematical function or visual inspection.	Incremental stages: (i) using all stages analyzing by visual inspection; (ii) using all stages analyzing by 2° order polynomial fit; (iii) using the first, second, and third stages (2° order polynomial fit); (iv) using the first, third, and sixth stages (2° order polynomial fit); (v) using the first, fourth, and sixth stages (2° order polynomial fit)	(i) 199.4 ± 19.4; (ii) 200.3 ± 19.1; (iii) 199.8 ± 19.4; (iv) 202.0 ± 18.5; (v) 200.9 ± 19.0 m min^−1^. *P* > 0.05^#^; SMD-0.137.	(a) The LMI determination is not affect by using all or three incremental stages; (b) such results are not affected by mathematical function or visual inspection.
Pardono et al., 2008/Brazil.	11/recreationally cyclists / male.	3/to investigate the effects using all or only three incremental stages for LMI determination.	Incremental stages: (i) using all stages; (ii) three: first, stage based on RPE13 and last stage; (iii) three stages based on the lowest [lac]; (iv) initial, RPE13 and RPE16 stages. *P* > 0.05^#^; SMD-0.367.	(i) 188.5 ± 20.9; (ii) 183.6 ± 18.1; (iii) 190.4 ± 12.9; (iv) 192.1 ± 27.2 W	The LMI determination is not affect by using all or three incremental stages.
Johnson and Sharpe, 2011/United Kingdom.	8/recreationally active/male.	3/to investigate the effects of manipulating the initial intensity in the phase 3.	Initial intensity protocols: (a) 40% of the maximal power achieved from GXT performed on phase 1. (b) 45% of the maximal power achieved from GXT performed on phase 1.	(i) 175.0 ± 29.0 ; (ii) 184.0 ± 30.0 W *P* < 0.01^#^; SMD-0.305.	Despite the LMI determination being modified by initial intensity in the phase 3, the effects on LMI are small.
Miyagi et al., 2013/Brazil.	12/active male.	3/to investigate the effects using all or selected incremental stages for LMI determination.	Incremental stages: (i) using all stages; (ii) using all stages before and only two after LMI; (iii) using two stages before and all after LMI; (iv) using similar number of points and the highest possible number of stages before and after LMI; (v) using all points and also considering the blood lactate peak obtained on phase 2.	(i) 138.2 ± 30.3; (ii) 139.1 ± 29.1; (iii) 135.3 ± 14.2; (iv) 138.6 ± 20.5; (v) 136.7 ± 28.5 W. *P* > 0.05^#^; SMD-0.165.	The LMI determination is not affect by using all or selected incremental stages.

Note that the country was considered as the institution that authors participated in the publication year; SMD-standardized mean difference; AT-anaerobic threshold; GXT-graded exercise test; LMI-lactate minimum intensity; RPE-rate of perceived exertion;

# specific P-value was not provided;

§ studies may have instigated additional factors besides methodological concerns;

§§ *studies may have provided additional conclusions besides methodological concerns*.

### Phase 1: The role of the hyperlactatemia induction

As previously explained, subjects must initiate phase 3 of the LMT (i.e., incremental test) with high blood lactate concentrations. Therefore, the hyperlactatemia induction in phase 1 is indispensable. This necessity drew the attention of researchers regarding the possible influence that different hyperlactatemia modes may have on the LMT parameters. Smith et al. (2002) were pioneers in this area, showing that LMI determination was not dependent upon the hyperlactatemia induction protocols. Despite this interesting result, the authors pointed out the necessity of considering other methodological aspects rather than creating a hyperlactatemia state, such as the starting intensity and the workload increment in phase 3. Conversely, Zagatto et al. (2014) criticized the Smith's study mainly based on the 1 min exercise stage used. According to these authors, the 1 min stage is not sufficient to allow muscle-blood lactate efflux, underestimating the blood lactate concentration to its respective intensity. With a similar approach, Zagatto et al. (2014) proposed the effects of the hyperlactatemia induction protocol on LMI seems to occur when very strong exercises are used.

Studying the LMT phase 1 with a distinct perspective, Johnson et al. (2009) showed that the LMI determination is altered if different muscle groups are used during the hyperlactatemia induction. Active subjects performed two LMTs distinguished by phase 1, in which a GXT was performed in cycle (test 1) or arm-cranking (test 2) ergometers. Phase 3 was not altered and in both cases was applied in the cycle ergometer. The differences that were found regarding the LMI (test 1–168.0 ± 21.0; test 2–157.0 ± 29.0 W) were attributed by the different patterns in terms of lower lactate accumulation and greater oxidation presented in phase 3 by subjects that performed the hyperlactatemia induction in the cycle ergometer. These factors right-shifted the blood lactate kinetics in phase 3, increasing the LMI. Despite the novelty, these findings did not substantially improve the possible phase 1 influences on LMI determination once the dependence of the AT achievement (which can be extended to the LMI) on the motor pattern of exercise was already found (Beneke and von Duvillard, 1996). Therefore, the difference in terms of LMI shown by Johnson et al. (2009) should be carefully considered, since it does not demonstrate that phase 1 affects the LMI determination, but advocates that specificity regarding the motor pattern should be consistent throughout the LMT phases. Despite the efforts conducted to investigate the influences of hyperlactatemia induction on LMT parameters, investigation of this issue is still required. As Zagatto et al. (2014) have criticized a methodological question in the incremental test used by Smith et al. (2002) (i.e., 1 min stage length), we can highlight other methodological points that are missing in both studies. Firstly, the LMT proponents did not stipulate that the hyperlactatemia induction must result in a certain blood lactate concentration, since subjects may respond individually to the high intensity effort performed in phase 1. Therefore, subsequent studies can investigate whether this phase results in a peak blood lactate concentration or if it is only necessary to elevate the serum lactate concentration without reaching its peak. Can the peak attainment be dependent on the hyperlactatemia mode? What is the influence of peak blood lactate level attainment on LMT parameter determination? The rationale beyond the aforementioned questions is grounded in the fact that the blood lactate peak concentration was recently proposed to determine the LMI in phase 3 (Miyagi et al., 2013).

To answer these questions, future studies can compare two LMT applications (and its results) in which subjects initiate phase 3 and do or do not attain its blood lactate concentration peak from phase 1. It is valid to state that the main goal of phase 1 is to achieve the hyperlactatemia state. In this sense, the recovery that is subsequently required on phase 2 may influence the answers from the proposed questions (see next Section). Thus, further studies with the purpose of answering these questions should also consider the methodological concerns of phase 2. These will be discussed in the next subsection.

### Phase 2: Active or passive recovery? what about the intensity and length?

Another LMT methodological concern lies on the recovery manipulation after the hyperlactatemia induction. Despite the fact that several studies followed the original 8 min recovery (Bacon and Kern, 1999; Carter et al., 1999a,b, 2000; MacIntosh et al., 2002; Dantas De Luca et al., 2003; Ribeiro et al., 2003; Zagatto et al., 2004, 2013, 2014; Simões et al., 2005, 2009; Hiyane et al., 2006; Altimari et al., 2007, 2010; Azevedo et al., 2007; Silva et al., 2007; Pardono et al., 2008; Johnson et al., 2009; Johnson and Sharpe, 2011; Mota et al., 2011; Barbieri and Gobatto, 2013; Camargo et al., 2013; Mezzaroba and Machado, 2013; Miranda et al., 2013; Miyagi et al., 2013, 2015; Campos et al., 2014; Messias et al., 2015), a myriad of recovery manipulations is also observed, especially regarding the recovery type and intensity (Davison et al., 2000; Smith et al., 2002; Rotstein et al., 2007; Silva et al., 2007; Sotero et al., 2007, 2009, 2011; Knoepfli-Lenzin and Boutellier, 2011; Vicente-Campous et al., 2014; Browne et al., 2015). Some attention has been given to this phase due to its potential LMI influence. For instance, Carter et al. (1999b) suggest whether the blood lactate was more quickly removed by an active recovery, then the LMI may be decreased due to a blood lactate concentration left-shift in the second order polynomial fit (i.e., “U” shaped).

Aiming to improve the knowledge on this topic, Ribeiro et al. (2009) submitted trained runners (regional level) to three identical treadmill LMTs, excluding the recovery phase. Confirming the Carter et al. (1999b) hypothesis, a decreased LMI was observed when a stronger active recovery (i.e., iii) was applied (i −13.8 ± 1.6; ii −13.3 ± 1.6 and iii −12.8 ± 1.5 km.h^−1^). Notwithstanding, the HR, VO_2_, %VO_2max_ at LMI and the time to achieve the LMI also decreased when the recovery at 50% of the VO_2max_ velocity was used. These authors point out that the high intensity active recovery accelerates the blood flow and probably increases the metabolic rate of oxidative lactate tissues (e.g., heart and skeletal muscle), resulting in lactate clearance by an intensity-dependent manner. Additionally, Ribeiro et al. (2009) conclude that the LMI among other results seem to be dependent on the recovery adopted, especially when high intensity active recovery is used.

Despite the advances related to the recovery type and intensity, some concerns were not investigated so far, especially in terms of recovery length. The main goal of this phase is to provide sufficient time for the lactate transposition from cells to the bloodstream. However, is the 8 min recovery proposed by LMT proponents the most appropriate length for this phase? From our point of view and others (Carter et al., 1999b), the 8 min standardization for all subjects can result in sub- or super-estimation of the time required for the blood lactate transposition. We again confirm that the “U” shaped form during the LMT is expected, considering subjects will initiate phase 3 with high blood lactate concentration. Subsequently, considering the low intensity of the first incremental stages, the blood lactate is expected to fall into the minimum point and to rise again during the latter incremental stages. However, the “U” shaped form cannot occur if the recovery was beyond (super estimation) or insufficient (sub estimation) when compared to the real subject's necessity. In the super-estimation case, phase 3 can be initiated with low blood lactate concentrations, since the lactate clearance has already occurred during phase 2. Therefore, the “J” form can be obtained instead of the “U”. In the sub-estimation case, subjects can perform the first incremental stages and still result in a high blood lactate concentration, since the given time for phase 2 was not long enough for the lactate transposition; theoretically, in this case, the “U” shape and the minimum point could be right-shifted.

The recovery length can be influenced by aerobic capacity and sports specificity (e.g., endurance or sprinters runners; Carter et al., 1999b; Denadai and Higino, 2004). Literature has demonstrated that lactate clearance is improved in aerobically trained individuals (Donovan and Brooks, 1983) via lactate transporters known as monocarboxylic acid transports (MCTs; Juel and Halestrap, 1999; Halestrap, 2013). High cellular expression and content of MCTs is expected in such individuals (Thomas et al., 2012). Another fact that corroborates the role of MCTs on lactate clearance in trained individuals is the improved lactate utilization as energy is used in tissues or organs that are not directly involved during exercise (Brooks, 1986; Glenn et al., 2015). Therefore, from a biomolecular point of view, the lactate clearance in LMT phase 2 can be influenced by the MCT activity, which is turn, is related to the aerobic capacity. Although, the rationale of these questions seems plausible, the relationship between MCT activity and the blood lactate clearance on phase 2 requires investigation.

Further discussion related to the LMT phase 2 is still required. We have suggested that the 8 min cannot be adequate for all subjects. However, whether the determination of LMT parameters depends on the recovery length is open for investigation. Evidence has supported that the LMI determination is not a function of standardized or individualized recovery during phase 2 (Denadai and Higino, 2004), but further investigation around this issue was requested. Subsequent studies can examine this question by applying the LMT with a standardized recovery length (i.e., 8 min) and an individualized one. The physiological characteristics of subjects must also be accounted for. As previously suggested (Denadai and Higino, 2004), this allows for investigation around several ranges of recovery time, including recovery times shorter than 8 min. In the individualized protocol case, a previous test will be required. During the first evaluation, the same hyperlactatemia mode that will be applied in the LMT must be conducted. Then, by collecting blood lactate samples every 30 s after the hyperlactatemia induction, researchers may determine the moment in which the blood lactate concentration reaches its peak. This will identify the individual recovery length. Under the same laboratory conditions, the LMT can be applied in the next evaluation section considering this individual length. This design can reveal whether the determination of LMT parameters depends on the recovery length.

### Phase 3: Implications on the incremental test

The last concern about the LMT methodology is related to the incremental test (phase 3). Among other evaluations, the incremental test is a valid procedure for determining submaximal and maximal variables. Nonetheless, it is evident in scientific studies that the greatest variation is a result of the type (i.e., stage length and duration) of incremental test used (for review see Bentley et al., 2007). The same variation is also observed in studies involving the LMT application (Bacon and Kern, 1999; Carter et al., 1999a; Davison et al., 2000; MacIntosh et al., 2002; Ribeiro et al., 2003; Johnson and Sharpe, 2011). As previously explained, the LMT proponents have already shown concerns about such issues. However, Carter et al. (1999b) criticize the original study by suggesting that the lactate production and its efflux to the bloodstream are intensity and time dependent. In addition, these authors also suggested that the starting incremental test intensity may have an important role in determination of the LMI since the blood lactate kinetics could be directly influenced if different starting intensities are used. Indeed, Tegtbur et al. (1993) applied the initial intensity based on the “individual recovery speed,” but these authors did not clarify why this intensity was used.

Aiming to investigate this issue, Carter et al. (1999b) submitted endurance runners to eight similar LMTs except for the incremental starting intensity, which ranged from 3.0 km.h^−1^ below the AT_2_ to 1.0 km.h^−1^ above the AT_2_. These authors demonstrated that the LMI is affected by the starting intensity of the incremental phase, since lower starting intensities resulted in decreased LMI and vice-versa, with the exception of the intensities at the AT_2_ and 1.0 km.h^−1^ above that made it impossible to determine the LMI. These results are partially explained by the fact that at low exercise intensities the lactate removal from the bloodstream is enhanced by visceral organs (e.g., kidney, heart, and liver) and at higher intensities there is cardiac output redistribution away from viscera to supply the blood demand of working muscles. Since the incremental starting intensity directly affects the blood lactate kinetics, the authors conclude that the LMT is an invalid protocol due to its dependency on LMI determination. Even though Carter et al. (1999b) suggested that the LMI determination may also be influenced by the mathematical procedure employed, this topic was not examined. Additionally, the effect of the stage length was also overlooked by these authors.

Four years later, Ribeiro et al. (2003) investigated the effects of both stage length and mathematical procedures on LMI determination. Evaluating trained swimmers (national level), these authors showed that the LMI was not affected by different stage lengths (200 or 300 m) or the mathematical procedure employed (visual inspection or spline function), at least for swimming exercises. These results partially corroborate with the original study of Tegtbur et al. (1993), which showed that the LMI is not influenced if 800 m or 1200 m distances were applied in the incremental running test. The novelty provided by Ribeiro et al. (2003) lies in the independence of the mathematical procedure employed for LMI determination, since low errors of 0.05 and 0.01 m.s^−1^ were obtained when using the visual inspection or spline function, respectively.

Despite the fact that Sotero et al. (2007) raised the LMI determination possibility within reduced blood samples, Pardono et al. (2008) investigated whether less incremental stages or indirect methods could be used for estimating the LMI by polynomial functions in recreational cyclists. These authors showed that the LMI based on the rate of perceived exertion (LMP_3sub_) or the three lowest blood lactate stages (LMP_3adj_) were not different from the iMLSS (LMP_3sub_ −192.1 ± 27.2; LMP_3adj_ −190.4 ± 12.9; iMLSS −204.0 ± 16.0 W), proposing alternative or shorter forms for LMI determination. The shorter version of the LMT was also studied by Simões et al. (2009), which showed no difference between the LMI determined by visual inspection and several polynomial mathematical functions, including the utilization of only three selected stages and a novel lactate quotient equation (i.e., LMVQ-blood lactate concentration velocity^−1^ ratio plotted against the correspondent velocity). Positive results regarding the LMI determination within less incremental stages were also published by recent investigations (Sotero et al., 2011; Miyagi et al., 2013).

Based on the aforementioned literature, it is clear that efforts were made to analyze methodological concerns in phase 3. However, future investigations are still required, mainly based on stage length and intensity. These factors can be discussed along with a novel and recent evaluation protocol entitled the reverse lactate threshold (RLT) proposed by Dotan (2012). In this new proposal, the AT is identified in reverse, since after three warm-up stages (~4 min length) with sub AT intensities, a supra AT effort is performed; thereafter, subsequent stages with sub AT intensities are again conducted to identify the highest intensity in which lactate production and clearance are balanced. Although this new proposal is not directly related to the issues of this section, some concerns highlighted during the RLT can be transposed to the LMT application, mainly regarding the stage length and intensity.

Dotan (2012) claims that <3 min duration stages can underestimate the AT intensity, since short lengths do not enable enough time for lactate transposition from cells to the bloodstream. On the other hand, long-duration stages (>5 min) will result in high blood lactate accumulation, impairing the real AT determination. Regarding the intensity, the author also suggests that if high intensity efforts were conducted (mainly in supra AT intensity stage during RLT), the subjects could reach premature exhaustion. These two concerns highlighted during the RLT should be transposed for LMT application. Despite the fact that Ribeiro et al. (2003) showed that the stage length does not influence LMI determination, we show that the length in this study was considered in terms of distance. Considering the study by Dotan (2012), future investigations should compare several LMT applications of different stage lengths with fixed time and investigate if LMI is influenced by this factor. This perspective was already explored in GXT applications and its influence was already demonstrated (Yoshida, 1984). Regarding the stage intensities, a novel investigation should be conducted by comparing two LMT applications in which stage intensities are based on AT from alternative protocols (e.g., GTX) (test 1) or based on iMLSS (test 2). Since the MLSS is the gold standard protocol for AT determination, the LMI reliability should be demonstrated in the latter test. Furthermore, in order to improve LMI precision we suggest that small intensity increments between stages (5%) should be applied.

## Is the LMI modified after training?

The relevant studies involving training effects on LMI in humans are shown in Table 2. Carter et al. (1999a) were the first to investigate the effects of training on the LMI. These authors suggested that this topic must be properly studied to prove whether the LMT is useful for longitudinal monitoring of training effects. Thus, by evaluating sports science students, this study showed that the LMI did not change after 6 weeks of endurance training (before −11.0 ± 1.7; after −10.9 ± 1.7 km.h^−1^), despite significant improvements in VO_2max_ (before −3.3 ± 0.7; after −3.6 ± 0.8 l min^−1^), AT_2_ determined during the incremental test (before −11.2 ± 1.8; after −11.9 ± 0.8 km.h^−1^), iMLSS (before −13.3 ± 1.7; after −13.9 ± 1.6 km.h^−1^) among other variables. Based on these results, Carter et al. (1999a) concluded the LMT is not capable of identifying longitudinal effects of endurance training, at least when the identical LMT is applied before and after the training.

**Table 2:** Characterization of studies with humans that investigated the effects of longitudinal training on the lactate minimum intensity (LMI).

**Study authors/country**	**Study population (n°/training level/ gender)**	**Study aim^§^**	**Study results—LMI before/after**	**Study conclusion^§§^**
Carter et al., 1999a/United Kingdom.	24 (16 completed the training/8 acted as non-training control)/recreationally active (*n* = 15); highly trained (*n* = 1)/13 males and 11 females.	To investigate the LMI sensitivity to six weeks of endurance exercise training.	11.0 ± 0.7/10.9 ± 1.7 km.h^−1^ *P* > 0.05^#^; SMD-0.083.	Since other parameters significantly changed after the training period (e.g., iMLSS), it was concluded he LMI is not sensitive for identifying longitudinal training effects.
Silva et al., 2007/Brazil.	13/soccer athletes/male.	To investigate the effects of eight weeks of soccer training on the LMI.	14.9 ± 0.2/15.4 ± 0.4 km.h^−1^ *P* < 0.05^#^; SMD-1.667.	The eight weeks of soccer training resulted in positive improvements of the LMI.
Miranda et al., 2013/Brazil.	13/soccer athletes/non-specified.	To investigate the effects of ten weeks of soccer training on several parameters (i.e. anthropometric, psychological, skill) including the LMI.	9.9 ± 0.5/11.2 ± 0.6 km.h^−1^ *P* < 0.05^#^; SMD-2.364.	The ten weeks of soccer training resulted in positive improvements of the several parameters analyzed, including the LMI.
Campos et al., 2014/Brazil–Denmark.	8/trained swimmers/4 male and 4 female.	To investigate the effects of twelve weeks of swimming training on the LMI.	Swimmers were evaluated in three moments: (i) before training–1.1 ± 0.1; (ii) after four weeks–1.1 ± 0.1; (iii) after twelve weeks–1.2 ± 0.1 m.s^−1^ *P* = 0.003 (i ≠ ii and iii); SMD-1.000.	The LMI was partially changed during the twelve weeks of training, since no modification was found between four and twelve weeks (ii = iii).
Manunzio et al., 2016/Germany.	5/experienced cyclists and triathletes (*n* = 4) and backup athlete (*n* = 1).	To add a detailed physiological and performance profile for ultra-endurance athletes over their six month preparation period for an 4,800 km non-stop cycling race.	Athletes were evaluated in three moments in terms of LMI (besides other evaluations): (i) before preparation–272.0 ± 48.0; (ii) after three months–282.0 ± 34.0; (iii) after six months–292.0 ± 50.0 W *P* = 0.010 (i ≠ iii); SMD-0.408.	LMI increased significantly over the training period.

Note that the country was considered as the institution that authors participated in the publication year; SMD-standardized mean difference; LMI-lactate minimum intensity; iMLSS-maximal lactate steady state intensity;

# specific P-value was not provided;

§ studies may have instigated additional factors besides training effects;

§§ *studies may have provided additional conclusions besides training effects*.

Conversely, Silva et al. (2007) showed that the LMI of elite soccer athletes significantly improved after 55 sessions (i.e., 8 weeks) of endurance, anaerobic, agility, coordination, strength and sprint training (before −14.9 ± 0.2; after −15.4 ± 0.4 km.h^−1^) sessions. Even with a different initial subject training status, frequency and training load, significant LMI improvements were found for youth soccer athletes after 10 weeks (before −9.9 ± 0.5; after −11.2 ± 0.6 km.h^−1^) (Miranda et al., 2013), youth swimmers after 12 weeks (before −1.1 ± 0.1; after −1.2 ± 0.1 m.s^−1^) of training (Campos et al., 2014) and also over a 6 month preparation period of ultra endurance athletes (Manunzio et al., 2016).

Although, these results corroborate the LMT sensitivity to monitor longitudinal training effects, a limitation already addressed by Carter et al. (1999a) was highlighted. Considering the fitness improvement after the training, the intensity of the incremental stages must be modified after the training period. Otherwise the identical LMT applied after the training could underestimate the actual participant physiological condition. This will lead to a fast blood lactate removal in the first incremental stages, resulting in a left-shift of the second order polynomial fit.

This issue must be carefully investigated, since it is possible that there is a mathematical-dependency on the LMI determination and therefore its utilization for longitudinal monitoring of training effects can be dubious. Carter et al. (1999a) determined the LMI from nadir of the spline function between blood lactate and running speed. If after training there is a fast blood lactate clearance due to the fitness improvement, then the blood lactate concentration during the first incremental stage should be left-shifted. When the spline function is applied, the LMI is determined considering the nadir (i.e., the lowest point of the spline function). This mathematical analysis could lead to a misleading LMI determination, as it will consider the first minimum point and disregard the other points. After training, the “U” shaped form and the nadir will be more pronounced, considering that more stages could be performed by subjects whether aerobic improvements are achieved during the training period or not. In addition, the stage intensity after training could not be same when compared to before. The lactate can be removed quickly (i.e., clearance), but the subject may also sustain the subsequent incremental stages producing less lactate, resulting in an aerobic improvement. In this case, a second polynomial fit should be preferred, since the LMI determination will be identified from the derived equal zero of the polynomial equation (considering all points) and therefore right-shifted.

The aforementioned hypotheses remain to be tested. Factors such as the type/load of training as well as the subject's characteristics should be taken into consideration. Some studies provided evidence that the mathematical dependency on LMI determination after some training periods may occur. For instance, improvements in terms of LMI were not observed when the spline function was applied (Carter et al., 1999a), but significant modifications of this parameter were obtained when the second order polynomial was used (Silva et al., 2007; Miranda et al., 2013). A novel study is still required to investigate whether the mathematical dependecy on LMI after training indeed exists. Such studies should consider that subjects must present improvements after the training period, otherwise the mathematical dependency could not be investigated. Along with the LMT application, we suggest that future studies also conduct the MLSS protocol to investigate whether improvements will indeed occur. Despite the fact that the experimental design of Carter et al. (1999a) covers this question, the mathematical dependecy was overlooked and our hypothesis is therefore still open for testing.

## Does the LMI indeed estimate the iMLSS?

The MLSS is considered the most accurate protocol for AT determination. To precisely determine the iMLSS, at least 4–5 evaluation days are suggested (Billat et al., 2003). The myriad of definitions for iMLSS determination is also observed in scientific studies. While authors use different criteria regarding blood lactate increases (e.g., increases of 0.5 or 1.0 mmol.l^−1^) (Mocellin et al., 1990; Williams and Armstrong, 1991; Beneke and von Duvillard, 1996), others have considered different times to calculate the blood lactate increase throughout the test (Haverty et al., 1988; Aunola and Rusko, 1992; Urhausen et al., 1993). These methodological aspects were also criticized in the original LMT article (Carter et al., 1999a,b). Tegtbur et al. (1993) showed that a 0.2 m.s^−1^ increase on the LMI was sufficient to elicit significant increases of 1.9 ± 1.1 mmol.l^−1^ in blood lactate concentration after an 8 km.h^−1^ constant run. The fact is that the 0.7 km.h^−1^ increment can be a wide intensity range and therefore provide a misleading iMLSS estimation. It is possible that the first working hypothesis of Tegtbur et al. (1993) is correct, but subsequent studies are required to properly investigate this issue.

Jones and Doust (1998) were the first to investigate this topic by comparing the LMI and the iMLSS of well-trained runners. After applying the MLSS (i.e., five constant-velocity treadmill runs of 30 min duration) and the original LMT, these authors observed that the LMI (14.9 ± 0.2 km.h^−1^) was different (*P* ≤ 0.05) from the iMLSS (15.7 ± 0.3 km.h^−1^) and they were poorly correlated (*r* = 0.61). The authors drew two conclusions from the results: a) future investigations should consider methodological aspects in every LMT phase, since these factors could influence the LMI determination (for complete discussion see the previous section) as well as its comparison with iMLSS; and b) according to results the LMT has poor discriminatory power between subjects, since a low relationship was observed between the LMI and iMLSS.

Two years later, Bacon and Kern (1999) insisted on studying this topic by comparing the LMI and the iMLSS of active women. These authors used similar procedures to Tegtbur et al. (1993), except for the incremental test used to induce hyperlactatemia and the smaller 0.13 m.s^−1^ (~0.5 km.h^−1^) increment above the predicted LMI during a constant velocity 28 min run. In addition, the definition for steady state was fixed as a blood lactate concentration less than or equal to 0.5 mmol.l^−1^ between the 4th and 28th min. Thus, while 0.6 ± 0.3 mmol.l^−1^ was found in the constant run at the LMI, high increases of 1.8 ± 0.3 mmol.l^−1^ were obtained in the constant run at 0.13 m.s^−1^ above LMI. Based on the experimental procedures used, Bacon and Kern (1999) concluded that the LMI predicts the iMLSS. Such conclusions were also drawn by studies of cycling (MacIntosh et al., 2002; Knoepfli-Lenzin and Boutellier, 2011), running (Sotero et al., 2009), and swimming (Ribeiro et al., 2003) exercises.

Although, Knoepfli-Lenzin and Boutellier (2011) showed good agreement between the LMI and iMLSS (97% inside the limits of agreement) (Bland and Altman, 1986) and high correlation (*r* = 0.86), a slight but significant difference was also observed in cyclists (LMI = 245 ± 29 and iMLSS = 255 ± 32 W; *P* ≤ 0.01). These authors suggest this difference could be explained by the range of a 5 W intensity used for determining the iMLSS, which resulted in a 4% difference between the two intensities. At this point, we highlight a possible methodological concern in the iMLSS determination that could be influenced on the conclusion of the aforementioned study.

As previously noted in the beginning of this section, the myriad of MLSS applications can directly influence the iMLSS determination. Beneke (2003) suggests that the 0.5 mmol.l^−1^ difference between the 10th and 20th min provides low iMLSS when compared to the 1.0 mmol.l^−1^ difference between the 10th and 30th min. Although the discussion regarding the methodological aspects in the MLSS is beyond the scope of this study, we must highlight this fact since the comparison between the LMI and iMLSS could be directly influenced by this factor. This is relevant because studies that used 0.5 mmol.l^−1^ (Sotero et al., 2009) or 0.7 mmol.l^−1^ (MacIntosh et al., 2002) did not observe differences between LMI and iMLSS. On the other hand, when the 1.0 mmol.l^−1^ was considered (Jones and Doust, 1998; Carter et al., 1999a; Knoepfli-Lenzin and Boutellier, 2011; Perret et al., 2012) the iMLSS was significant higher than the LMI (Table 3).

**Table 3:** Characterization of studies with humans that compared the lactate minimum intensity (LMI) with the maximal lactate steady state intensity (iMLSS).

**Study authors/country**	**Study population (n°/training level/ gender)**	**Study aim^§^**	**Exercise type**	**Study results–LMI vs. iMLSS**	**Study conclusion**
Jones and Doust, 1998/United Kingdom.	13/well-trained/male.	To investigate the LMI validity to estimate the iMLSS.	Running	LMI – 14.9 ± 0.2 km.h^−1^ iMLSS – 15.7 ± 0.3 km.h^−1^ *P* < 0.05^#^; SMD-3.200.	Since differences were found between the two intensities, it was concluded that further experimental investigations are required before conclude the LMI estimates the iMLSS.
MacIntosh et al., 2002/Canada.	14/athletes–cyclists or triathletes/11 males–3 females.	To investigate the validity of the LMI as predictor of iMLSS.	Cycling	LMI – 33.6 ± 3.5 km.h^−1^ iMLSS – 33.5 ± 3.1 km.h^−1^ *P* = 0.900; SMD-0.030.	The LMI is a valid predictor of the iMLSS.
Johnson et al., 2009/United Kingdom.	32/active/male.	To evaluate the agreement between a LMI from a modified LMT with the iMLSS.	Cycling	LMI – 205.0 ± 22.0 W iMLSS – 208.0 ± 25.0 W *P* ≤ 0.05^#^; SMD-0.128.	There was good agreement between the LMI from a modified LMT with the iMLSS.
Knoepfli-Lenzin and Boutellier, 2011/Switzerland.	63/moderately to highly trained/male.	To investigate whether LMI is valid to estimate the iMLSS in subjects with different levels of fitness.	Cycling	LMI – 245.0 ± 29.0 W iMLSS – 255.0 ± 32.0 W *P* < 0.01^#^; SMD-0.328.	Despite the significant difference between the two intensities, it was concluded the LMI is valid to estimate the iMLSS, since the difference was small and high and significant relationship was found (*r* = 0.866; *P* < 0.01^#^).
Dotan et al., 2011/Canada–Israel.	16/trained runners/male.	To compare the LMI with iMLSS and re-evaluate the LMT dismissal.	Running	LMI was analyzed by visual inspection (LMIm) from two reviewers (mean) and 2° polynomial fit (LMIp); LMIm – 13.2 ± 1.0 km.h^−1^ LMIp – 13.0 ± 1.0 km.h^−1^ iMLSS – 13.5 ± 0.9 km.h^−1^ *P* = LMIm vs. iMLSS – 0.010; LMIp vs. iMLSS = 0.001; SMD-0.441.	The LMI underestimates the iMLSS; however, the nature of this difference is still unclear and further efforts are required.

Only studies that properly applied the maximal lactate steady protocol were included. Investigations that solely investigated whether the LMI correspond to iMLSS are discussed in the text. Note that the country was considered as the institution that authors participated in the publication year; SMD-standardized mean difference; LMI-lactate minimum intensity; iMLSS-maximal lactate steady state intensity;

# specific P-value was not provided.

§ *studies may have instigated additional factors besides comparison between LMI and iMLSS*.

The comparison between these two intensities is necessary for practical applications. Additionally, the requirement of several days for the MLSS application is not welcome in current sporting calendars. Instead, the LMT could be an interesting proposal to save time. In addition, few blood collections are required during the LMT, resulting in less discomfort for the subject and improving the test objectivity. In this way, alternative LMT methods to identify the iMLSS were proposed (Pardono et al., 2008; Sotero et al., 2009). However, considering the different results among scientific studies published so far, future efforts around this issue are required. A novel study should compare the LMI and iMLSS determined within different criteria for blood lactate increase, such as 0.5, 0.7, or 1.0 mmol.l^−1^. Such designs should confirm whether this methodological aspect of the MLSS protocol influence the comparison between these two intensities.

## Animal models and the LMT

Despite the fact that the LMT was originally proposed for running exercise, its application is not limited to humans. Studies successfully adapted this test for rodents (Voltarelli et al., 2002) and equines (Gondim et al., 2007). Additionally, several investigations involving animal models of different backgrounds were published. Some examples include methodological analyses (de Araujo et al., 2007; Beck et al., 2012; Sena et al., 2015), effects of periodized or non-periodized training (de Oliveira et al., 2007; de Araujo et al., 2012, 2013a, 2016; Scariot et al., 2016) effects of luminosity and time of day (Beck and Gobatto, 2013, 2016; Beck et al., 2014; Beck et al., 2014a,b) and comparion with the MLSS protocol (Rodrigues et al., 2016).

The first study that adapted the LMT for rodents was conducted by Voltarelli et al. (2002). Inside a cylinder tank (50 cm deep and 25 cm in diameter) filled with water at 31 ± 1°C, Wistar rats (*Rattus norvegicus*) were induced to jump into the water for 6 min (30 s of exercise and 30 s of rest) carrying loads relative to 50% of the body mass (i.e., load) on the chest to induce hyperlactatemia (phase 1). After 9 min of recovery (phase 2), the animals were submitted to an incremental swimming test (phase 3). The incremental test was composed of 5 min stages with loads relative to 4.5, 5.0, 5.5, 6.0, and 7.0% of the body mass. At the end of each stage (30 s), blood samples (25 μl) from the distal tail were collected for the blood lactate concentration analysis. By using a spline function, these authors observed the “U” shaped form proposed by Tegtbur et al. (1993), and the LMI was defined as the zero gradient tangent of the curve.

After the aforementioned study, several methodological analyses were conducted to investigate the reliability of the LMI determination in Wistar rats. For instance, the possible influence of phase 1 on the LMI determination was investigated in an animal model by de Araujo et al. (2007). Wistar rats were submitted to four similar swimming LMTs with the exception of the hyperlactatemia protocols, which were composed of different loads. These authors showed that the LMI was not significantly different from the iMLSS, independent of the hyperlactatemia protocol used. However, protocol 4 (protocol 4 consisted of a deep swim effort of 13% of the body mass and after 30 s of passive recovery, a second effort at the same intensity until exhaustion) was suggested, since it presented a high success rate for LMI determination (91% of the cases). To date, this study was the first to propose the success rate criteria for the LMI determination, which considered the presence of the “U” curve (i.e., polynomial fit) followed by a *R*^2^ ≥ 0.80. Regarding the mathematical models, Beck et al. (2012) found the LMI is not modified whether visual inspection, polynomial fit or spline models are used.

The LMT was also successfully applied in equines by Gondim et al. (2007). Previously the test, using five horses (Arab *n* = 2; 1/2 Q.H. *n* = 1; Anglo Arab *n* = 2) ridden by their owners, was a 500 m full gallop on a field track (1200 m, grass surface) for identification of the individual time that the peak lactate concentration is attained. After 36 h, the horses were submitted to the LMT, which was composed of the same 500 m full gallop to induce hyperlactatemia (phase 1), a recovery rest based on the individual time that the peak lactate concentration was attained (phase 2), and finally an incremental test was initiated at 15 km.h^−1^ with 2 km.h^−1^ increments at each 1000 m (phase 3). Blood samples from the jugular vein were collected at the end of the stages, and the LMI was most likely determined by visual inspection (this was not clarified in the study). After the LMT the animals were submitted to two 10000 m trials, and procedures that were similar to Tegtbur et al. (1993) were followed. The first trial was performed at the LMI and the second at 10% above the LMI. The authors observed that only the second trial resulted in significant lactate elevation above resting values. The authors conclude that the LMI estimates the iMLSS, but as in the original LMT study, the MLSS protocol was not properly applied. Until now, the concordance between the LMI and iMLSS in horses is not established, since two studies with different conclusions have been published on this issue so far (Miranda et al., 2014; Soares et al., 2014).

Considering the stress that several evaluation days impose on the animals, the LMT is an excellent option (Lima et al., 2017). Regarding the Wistar rats, the LMT is extensively used in different contexts as a valuable tool to estimate the aerobic capacity. In addition, authors have used the time to exhaustion (i.e., *T*lim), obtained during the second swim effort at 13% of the body mass (phase 1; de Araujo et al., 2007), as an anaerobic metabolism indicator (de Araujo et al., 2012, 2013a,b). Thus, the LMT is considered a complete evaluation for Wistar rats, forasmuch as in one aerobic and anaerobic test results can be achieved. Similarly, the LMT is an excellent procedure for horses. Gondim et al. (2007) highlight that endurance horse events have been gaining popularity around the world. This fact is requiring special attention to the AT determination from riders, since these animals might be involuntarily submitted to high extenuating efforts, sometimes leading to fatal consequences. Thus, as a simple and inexpensive protocol, the LMT is strongly recommended for these animals.

## Conclusion

The LMT popularity is noteworthy considering its application and data interpretation. The protocol introduced by Tegtbur et al. (1993) was recognized in the scientific community and has been extensively used in many exercises in humans and animal models. As discussed in this review, despite the LMT advantages such as time and cost saving, futures studies are required to improve the scientific knowledge around this test. Overall, this review highlighted questions that still require investigation. First, despite advances that have been made regarding methodological concerns of LMT application, several points are still missing and future studies are encouraged to deal with these questions. Second, little information was provided regarding the LMT effectiveness in identifying improvements for training. A novel investigation is required to address this issue, especially considering the possible mathematical dependency highlighted in this review. Third, efforts to prove the LMI as a valid iMLSS estimator were made; however, methodological aspects in both protocols have hampered this discussion. Further analysis to investigate these factors are worthy, since the LMT is an important physiological evaluation for cases in which exercise physiology is used.

## Author contributions

LM, CG, WB, and FM contributed to the conception and design of the work, revision of the intellectual content and analysis and interpretation of the review.

### Conflict of interest statement

The authors declare that the research was conducted in the absence of any commercial or financial relationships that could be construed as a potential conflict of interest. The reviewer FRS and handling Editor declared their shared affiliation, and the handling Editor states that the process nevertheless met the standards of a fair and objective review.
